# Bridging peer support and primary care in youth mental health: stakeholder perspectives on needs, key elements and integration challenges

**DOI:** 10.1080/17482631.2025.2588933

**Published:** 2025-11-25

**Authors:** Rianne Pellemans-van Rooijen, Mark Spigt, Floor P.M. Koonings, Tom Odink, Verena G. Noort, Thérèse A.M.J. van Amelsvoort, Sophie M.J. Leijdesdorff

**Affiliations:** a Department of Psychiatry and Neuropsychology, Mental Health and Neuroscience Research Institute (MHeNs), Maastricht University, Maastricht, The Netherlands; b Department of Family Medicine, CAPHRI Care and Public Health Research Institute, Maastricht University, Maastricht, The Netherlands

**Keywords:** Peer support, youth mental health, primary healthcare integration, lived experience

## Abstract

**Introduction:**

This study explored how youth with mental health issues, peer support workers, and primary care professionals perceive youth’s needs while receiving support, key elements of peer support, and its integration into primary healthcare.

**Method:**

Twenty-eight semi-structured interviews were conducted and analyzed thematically to identify youth’s needs, core components of peer support, and collaboration challenges between peer support services and primary care.

**Results:**

All stakeholder groups recognized peer support as a valuable, low-threshold complement to traditional care, particularly for those awaiting treatment or lacking social support. Youth emphasized needs for knowledge, destigmatization, validation and connection - needs specifically addressed by the informal, empathetic, and non-hierarchical nature of peer support. Peer support workers and professionals emphasized the need to balance authenticity with safety and professional boundaries. Effective integration requires structured referral pathways, clear communication channels, confidentiality safeguards, and role clarity. Divergent expectations around responsibility for information sharing and formal requirements on lived experience highlight areas for improvement. Flexible peer support options that match youth's changing needs were seen as essential.

**Discussion:**

While peer support services offer a valuable addition to youth mental healthcare, its integration with primary care remains complex and requires improved communication, role clarification, and adaptable support options.

## 1. Introduction

Approximately 62.5% of adult mental health conditions originate before the age of 25 (Solmi et al., 2022), coinciding with critical periods of social, physical, and cognitive development (Kessler et al., 2005, 2007). The duration of symptoms during adolescence is particularly pivotal, as longer periods of untreated mental health issues strongly predict greater severity of mental disorders in later life (De Girolamo et al., 2012; Patton et al., 2014), contributing significantly to the global burden of disease (Rehm & Shield, 2019). Early intervention is therefore essential—not only to mitigate individual mental health issues but also to reduce long-term societal costs. One promising, yet underutilized, strategy in this context is peer support. This study explores how peer support can be effectively integrated into the primary healthcare system to better align its services with youth’s needs.

Despite the urgency, only 30% of young people in the Netherlands receive appropriate support for their mental health issues and the younger the individual, the more frequently unmet care needs are reported (De Graaf et al., 2010). On the other hand, not everyone requires specialized care, yet many still end up on long waiting lists. Within the Dutch healthcare system, general practitioners (GPs) are typically the first point of contact for youth with mental health issues. GPs play a vital role as gatekeepers to specialized services. Recent reforms aim to strengthen the role of primary care, given its accessibility, the high costs associated with specialized care, and the struggles of the specialized system to meet the increasing demand for mental health services (Inspectie Gezondheidszorg en Jeugd [IGJ], 2021; Van Yperen et al., 2019). Practice-based mental health professionals (POH-GGZs) can provide brief interventions with short waiting times, yet GPs and POH-GGZs face barriers such as limited time, difficulties with identification of mental health issues due to a lack of knowledge and skills, financial restrictions and difficulties with referrals and collaborations with other stakeholders (O’Brien et al., 2016). From the perspective of youth, stigma and a lack of knowledge about mental health issues, treatment options, waiting lists and costs remain significant obstacles for help-seeking (Leijdesdorff et al., 2021). Strengthening primary care-based mental health services and fostering interdisciplinary collaboration are critical steps toward addressing these systemic challenges and avoid service gaps at the vulnerable transition age of 18.

A key goal is to better align mental health services with youth’s needs while minimizing additional strain on overburdened primary and specialized care. One proposal is to reduce reliance on professional expertise by implementing peer support. Peer support is defined as a system of mutual assistance founded on respect, shared responsibility, and empathy, often provided by individuals with similar lived experience (Mead et al., 2001). It can take many forms, including one-on-one interactions, group settings, online platforms, walk-in services, or structured programs (Kent, 2019). It allows youth to share their experiences, feel less isolated, and receive practical guidance from trained peers, fostering resilience and personal growth (Castelein et al., 2008; Leijdesdorff et al., 2021; Mahlke et al., 2014; Shalaby & Agyapong, 2020). Peer support can complement or, in some cases, substitute specialized care, improving accessibility and potentially easing system burden (Boumans et al., 2023). Previous research suggests that peer support initiatives have consistent, though modest, effects on both personal and clinical recovery from diverse mental health issues (Richard et al., 2022; Smit et al., 2023; White et al., 2020). Moreover, these initiatives have been identified as a viable alternative for individuals facing barriers in accessing traditional care (Leijdesdorff et al., 2021).

While there is a growing body of research on the benefits of peer support, questions persist regarding effective implementation, particularly within primary healthcare settings. GPs and POH-GGZs expressed enthusiasm for integrating peer support into their practices, yet they reported lacking both the time and knowledge to implement such programs independently (Wix & Spigt, 2023). Further research is needed to support effective collaboration between peer support and primary care. First, research has yet to fully explore youth’s perceptions of peer support and their own specific needs within such programs. Second, knowledge regarding the perspectives of peer support workers and primary care professionals on the nature of peer support and how it complements primary care remains insufficient. Through semi-structured interviews, the perspectives of these three distinct stakeholder groups on youth’s needs in receiving care, as well as their views on key elements of peer support, will be systematically mapped in this study. Additionally, the study will explore how these stakeholder groups perceive the integration of peer support initiatives within the primary healthcare system, providing valuable insights for the effective collaboration between peer support and primary care.

## 2. Materials and methods

### 2.1. Study design

This study employed a qualitative research design with a phenomenological approach (Creswell & Poth, 2016) to explore youth’s needs and key elements of peer support from multiple stakeholder perspectives. Three separate interview guides were developed for interviewing youth, peer support workers and primary care professionals (see Supplemental Material A). These guides were informed by literature on peer support and youth mental health, as well as the research team's expertise. The interviews covered three main topics: (1) participants’ perspectives on the needs of youth with mental health issues when receiving support, (2) reflections on key elements of peer support, and (3) views on potential collaboration between peer support services and primary care.

### 2.2. Participants

Participants were recruited in multiple ways. There were no formal inclusion criteria other than proficiency in either Dutch or English and youth were required to have current or past mental health issues. Familiarity with or experience in peer support was valued but not required. Youth aged 12 to 25 were recruited through various channels, including GP practices, peer support locations, and informational leaflets distributed at educational institutions, such as university faculties, universities of applied sciences, and secondary vocational schools. The group of peer support workers consisted of volunteers from @ease locations in Heerlen and Maastricht. @ease is a Dutch peer support initiative offering youth aged 12 to 25 the opportunity to discuss their (mental health) concerns with trained young adult peers - often with lived experience - without requiring a prior appointment (Boonstra et al., 2023). Primary care professionals were mainly recruited via the @ease network and via snowball sampling (Naderifar et al., 2017). This group included both GPs and practice-based mental health professionals, who support GPs by providing consultation, screening and triage, short-term treatment, and transitional care. Participant inclusion continued until no new themes emerged during the interviews.

### 2.3. Study procedure

The study was reviewed by the Medical Ethical Review Committee (METC) of Maastricht University Medical Center and considered to not be subject to the Medical Research Involving Human Subjects Act (file ID: METC 2018−0961 & METC 2018−0961-A−9). All participants provided written informed consent. For participants under the age of 16, written informed consent was obtained from the parents, and written assent was obtained from the child. Youth received a gift voucher in appreciation for their participation.

Prior to the interviews, participants received background information about peer support to ensure a basic understanding of the concept (see Supplemental Material B). Interviews with youth and professionals were conducted by two members of the research team, while interviews with peer support workers were conducted by another researcher at a later timepoint. A semi-structured interview guide was used, with prompts included to give direction when needed. All interviews were recorded and transcribed verbatim. Interview locations varied based on participant preference, including GP offices, university facilities, @ease locations, or online. The duration of the interviews ranged from 33 to 68 minutes. Three interviews with youth and two interviews with peer support workers were conducted in English; all other interviews were held in Dutch.

### 2.4. Data analysis

Data analysis followed Braun and Clarke's thematic analysis framework (Braun & Clarke, 2006), using ATLAS.ti (version 23). The process began with data familiarization through transcription and repeated reading, followed by initial coding using both inductive and deductive strategies. The transcripts were analyzed in their original language (Dutch or English); they were not translated prior to analysis. Initial open coding of early transcripts was done independently by multiple researchers, followed by code comparison. Coding was conducted in English, and only excerpts from Dutch transcripts that were used as quotes in the manuscript were translated. Subsequent coding occurred with regular consultation within the research team to review coding decisions. Related codes were merged to develop themes, which were then refined and reviewed before final definition and naming. A comprehensive cross-stakeholder analysis was performed by comparing codes and themes across all three stakeholder groups to identify patterns, commonalities, and differences in perspectives.

## 3. Results

The results section is structured around four central themes. It begins with an overview of participant characteristics to contextualize the findings. Next, it explores youth’s needs in receiving support, based on perspectives from all stakeholder groups. This is followed by a description of participants’ perspectives on important key elements of peer support. Finally, the role of peer support in primary healthcare is discussed, with attention to opportunities and challenges in fostering collaboration between peer support services and primary care professionals. Illustrative quotes supporting the findings can be found in Table I.

**Table I.** Illustrative participant quotes by theme*.*

Theme		Quotes
1. Youth’s needs in receiving support	
* Perspective of youth themselves*	
Need for knowledge and control	– Y1: “To know what you have available itself is a challenge. That itself is a challenge. […] Sometimes you are not in the mind to look what is there.” – Y10: “Just which steps they took, what resolved it for them. And perhaps […] I can follow the same step-by-step plan.” – Y3: “I don't think anyone should say to me 'yes, you should do this now'. [...] That does not work with me. I need to be able to listen to someone, and I need to be able to take advice from them myself.”
Need for a healthy and normal development	– Y12: “I don't think like a doctor does it, who searches for a problem and looks for a solution, but just talking about your problems and what it does to you and so on. So it's not really a search for problems but just a pleasant conversation where you can share everything you're struggling with.” – Y8: “With a professional, you feel like they listen to you, but with peers, you feel like they truly understand what you’re going through.”
Need for validation and belonging	– Y12: “A [peer] could answer things and talk about it in a way a psychologist has never given me an answer. […] It was more about talking to someone who has experienced the same as you, a feeling of 'you're not alone, you're not an outcast or someone very different, there are others who feel this way and have been through this.”
Need for connection	– Y3: “Someone who doesn't necessarily have to provide answers, or what I should do now, but more someone who can just listen and to whom I can share my story.” – Y8: “I think I would rather just talk to people who had experienced the same, that they might not have been worried. That it was more just a free environment to discuss it and get help in a different way, instead of, using your normal [...] friends who eventually get a lot of worries in return, for what you tell them.”
* Perspective of peer support workers*	– PSW2: “What we do in the first place is listen, in second place you can advise them if they ask for it themselves.” – PSW3: “Here... it doesn’t matter what’s going on or who you are. There are no expectations.” – PSW7: “Many come because they don’t feel understood by their friends or family. They just want to be listened to without being judged. It’s important that they feel heard and respected, that alone can be healing.” – PSW7: “Openness, patience, and empathy are key. Not judging and not creating hierarchy is crucial.”
* Perspective of primary care professionals*	– Pro3: “I also think that we often need to normalize things. So instead of making it a problem, and maybe that can happen earlier if you say, 'Well, you don't have to go to the doctor or [...] just talk to someone there,' then I think you normalize problems earlier.” – Pro5: “Sometimes you also hear people say: ‘yes, I was a bit anxious, or I found it stressful to come and I even slept poorly because of it…’ whereas, I think if you’re dealing with a peer […], they don’t really feel that way because they see them as an equal person, yeah, just a regular person, a regular person, yeah.” – Pro2: “No, I don't think I've done that [provide information on options], not much. […] Yes, I also think that’s somewhat the responsibility of the patient themselves.”
2. Key elements of peer support	
* Informal and safe environment*	– Y7: “So I think taking that a little bit away from the healthcare center itself would be nice just to make it feel more implementable in your daily life and to reduce the barrier of going there.” – Y8: “I really like that you don’t need an appointment or an intake; you can just drop in when you need it.” – PSW1: “It feels homely at @ease… it doesn’t matter what your background or orientation is.” – PSW4: “You can take your shoes off here, that’s part of the vibe.” – PSW7: “So it's much easier to go for one conversation at @ease than to go to your GP and get in the records and get a psychologist and be on a waitlist and so on and so forth.”
* Peer support workers*	
Lived experience	– Y2: “I did feel that, even though I'm not in the same situation as them [peers], they were openminded and were quite understanding, which I haven't really found with other people my age.” – PSW1: “Sometimes it’s difficult to help someone who has gone through exactly the same thing, because it then triggers something in yourself. You also need to be stable yourself, otherwise you can’t handle helping others. […] I learn from it myself too, every single time. Everyone has a different story.” – PSW4: “Most volunteers have their own experiences with stress, depression, or anxiety. It helps to say: ‘I’ve been there too.’ You don’t need the exact same story to understand, empathy is enough.” – Pro1: “That it at least resembles each other strongly enough that you can put yourself in it. I know for example with autistic people, […] you could have someone who has had a panic disorder, but they can’t relate to what it’s like to feel different as a person among other people. That’s a different kind of feeling, different than with a panic disorder, so it really has to… yeah, it has to correspond enough.” – Pro5: “I don’t think that if someone comes here with burnout, which is not related to that trauma [of the peer], that you should let them come into contact with someone with trauma. I don’t think so, because then you’re actually going to trigger that trauma again, while that’s not what they came for.” – Pro2: “Yeah, you have to look at how that person is now relatively to what they have been through. Look, if they are still in the middle of it or, maybe because of that problem they’ve become embittered or […] not that realistic anymore […] then that could have negative consequences.”
Training and supervision	– Y8: “The peers are trained to provide support, which makes me feel safe and understood.” – PSW7: “Supervision is one of the best parts—it helps you learn and process difficult emotions. Sometimes you feel inadequate after a conversation, but supervision helps normalize that.” – Pro1: “Yes, I think that someone who is a peer should also be open to learning from what happens. So that person should also, yes, reflect on what has happened, and that you also… you do need a bit of guidance, someone who occasionally monitors things or gives feedback.”
Form of peer support	– Y13: “I think it would be pleasant to have the same person. [...] They slowly get to know you, and they also know more about you, they know what you've experienced before or what bothered you earlier. And if you have a new person every time, you have to tell your story again every time.” – Y2: “I think it could be nice if both scenarios are possible and that you can choose yourself what fits your need at that point, because I think there are a lot of different options out of which you could choose, which depends on the moment and the person’s medical condition. Everyone is different in that respect. […] so I think a lot of different available peers, that would be ideal.” – PSW2: “Online is low-threshold, but face-to-face is more effective.”
3. The role of peer support in primary healthcare	– Y7: “I wouldn't want the hierarchical thing. Not that I think there's a hierarchy between the patient and the doctor. […] But I think it would be nice to have someone that's exactly on your level […] that you feel very equal to, because I think that it's also easier to open up about things.” – PSW3: “I was on a waiting list for months. nobody told me about a place like this. Peer support could be really nice during that waiting period. It could be a bridge—before, during, or after therapy.” – PSW4: “It lowers the threshold for getting help—some wouldn’t go to a GP otherwise.” – Pro3: “Peer support is a good option for psychological problems that are not immediately pathological.”
* Cooperation between peer support and primary care*	– Y13: “For me it would not make a lot oof difference. But I would think to myself, if I have a peer support at the GP, then it’s like, that person can also not tell the doctor what I told them. So only with my permission, and if I say ‘no I don’t want you to say that’ then that won’t be told. Because that is not allowed, so for me wouldn’t be an issue.” – PSW7: “If GPs only say ‘go there’ without sharing private info, it keeps the threshold low. There could be some communication between GP, psychologist, and peer support—but only on a need-to-know basis.” – Pro2: “No, I actually prefer to refer someone only if I also know where I’m referring them to. Yes. I don’t like referring someone somewhere I know nothing about…” – Pro5: “Peers can be very helpful, but there should always be professionals available for situations that are too complex for peer support alone.”

Note: Y= youth, PSW = peer support worker, Pro = primary care professional.

### 3.1. Participant characteristics

A total of 28 participants took part in this study: fourteen youth, seven peer support workers and seven primary care professionals. The group of youth comprised individuals aged 14 to 26 (*M*_*age*_ = 21.4 years, *SD* = 3.52) with current or past mental health issues. Participants represented a heterogeneous sample with respect to mental health issues and demographic characteristics, including age, gender (five males, eight females, one non-binary individual), nationality (72% Dutch, 14% European, 14% non-European), and educational level (14.3% secondary school, 21.5% (higher) vocational education, 57.1% university level, 7.1% unknown). Of these people, 57% had no prior experience with peer support, 21.5% had experience with @ease, and 21.5% had engaged with another peer support initiative. Additionally, 57% of the participants were receiving specialized mental healthcare at the time of the interview. While most youth felt comfortable sharing their experiences, those further along in recovery were generally more reflective and better able to articulate their needs.

The group of peer support workers consisted of individuals volunteering at the @ease locations in Heerlen and Maastricht, comprising four males and three females aged between 22 and 34 years (*M*_*age*_ = 26.1 years, *SD* = 4.91). Their experience as volunteer at @ease ranged from 6 months to 6 years. Their interviews were analytical and reflective, connecting memories of their own hardships to the needs of @ease visitors and the importance of peer support.

The group of professionals included GPs (*n* = 3) and POH-GGZs (*n* = 4), comprising three males and four females. Two professionals had prior experience working directly with peer support workers, while three others had encountered patients who utilized peer support. The remaining two professionals reported no previous experience with peer support. Their interviews were interactive and reflective, with professionals expressing strong interest in the topic.

### 3.2. Youth’s needs in receiving support

#### 3.2.1. Perspective of youth themselves

Analysis of the interviews with youth revealed four key themes in their needs while receiving care: (1) knowledge and control, (2) healthy and normal development, (3) validation and belonging, and (4) connection. These needs, however, exhibit considerable variation both between individuals and over time, shaped by factors such as personal preferences and the severity of symptoms. The type of support required can thus fluctuate, depending on the evolving nature of youth’s mental health needs.

**Need for knowledge and control.** Youth highlighted the need for clear, accessible information about available care options, including lesser-known forms like peer support. Many searched for help online but often did not know what to look for. They expected healthcare providers - particularly GPs - to take a proactive role in introducing alternative forms of care. This would expand their awareness and enable them to make more informed choices. Being well-informed not only increased the likelihood of considering peer support, but also strengthened their sense of control over treatment decisions. Youth also valued guidance from those with similar experiences to help them navigate their own challenges while retaining the freedom to adapt advice to their own needs. Moreover, youth expressed a strong desire for control over treatment decisions, the type of support received, and how their personal information was shared, valuing a sense of ownership in their care. Despite limited experience with peer support, they viewed it as a flexible option that could meet their need for autonomy.

**Need for a healthy and normal development.** Youth wanted their mental health issues to be understood within the broader context of healthy and normal development, emphasizing the need for destigmatization, being taken seriously, and fostering a positive outlook. Many feared judgment due to persistent stigma, which made them hesitant to talk openly about their struggles. Peer support was perceived as a safe space for open dialog, where shared experiences reduce the likelihood of insensitive remarks and facilitate recovery. Participants also felt that being heard and acknowledged was crucial, as they often felt dismissed in traditional healthcare settings, leading some to question the legitimacy of their problems. Finally, they expressed a desire to focus on strengths and well-being rather than problems, seeing peer support as a positive, socially engaging alternative to problem-centered care.

**Need for validation and belonging**. Youth emphasized the importance of shared experience and a sense of resemblance to others facing similar challenges, as these provide validation, reassurance and self-acknowledgment. These factors were found to be more accessible through peer support than in interactions with friends, family, or healthcare providers. Equally important was the need for non-hierarchical, balanced relationships, which peers were seen as better able to provide than primary care professionals, fostering an open and welcoming environment for sharing experiences.

**Need for connection.** Youth identified connection as a crucial aspect of mental health support, encompassing social support, meaningful relationships, empathy, active listening, and reciprocity. They valued having a supportive presence during difficult times and saw peer support as a valuable alternative when personal networks were insufficient, especially in contexts of loneliness. Feeling understood - particularly from those with similar experiences - was key, as was the opportunity to talk openly and be genuinely heard. While some individuals appreciated reciprocal relationships where they could both give and receive support, others preferred the focus to remain on their own needs, illustrating diverse preferences.

#### 3.2.2. Perspective of peer support workers

The perspective of peer support workers closely aligned with the needs expressed by youth. Peer support workers emphasized their desire to offer perspectives and advice while maintaining a non-judgmental stance that respects young people’s autonomy. They aim to create an informal environment where experiences are normalized, open dialog is encouraged, and people feel heard and supported. Peer support workers recognized that their relationship with youth is less hierarchical compared to that between youth and healthcare professionals, which may foster more effective communication and support.

#### 3.2.3. Perspective of primary care professionals

Primary care professionals emphasized the importance of a patient-oriented approach, tailored to the needs expressed by youth, though they found it difficult to identify general needs. Nevertheless, they agreed that young people benefit from a familiar, trustworthy environment and valued peer representation as a way for youth to feel less alone. Additionally, professionals noted that a perceived imbalance in hierarchy between themselves and youth can hinder effective communication and support. While these views largely align with youth’s perspectives, a significant disconnect emerged regarding responsibility for accessing information about support options. Youth expected GPs to proactively share information on available treatment options, whereas some professionals place this responsibility on youth themselves, potentially creating a barrier to support in general and peer support in particular.

### 3.3. Key elements of peer support

The interviews revealed stakeholders’ perspectives on key elements of peer support. These include the creation of an informal and safe environment, the training and supervision of peer support workers, and the various forms in which peer support can be offered.

#### 3.3.1. Informal and safe environment

All stakeholder groups described peer support as an environment that should feel informal, relaxed, and clearly distinct from traditional healthcare, without a diagnostic focus. Key features for creating a safe space included confidentiality, familiarity and accessibility. Youth valued anonymity and the assurance that what is shared remains private. They also appreciated familiar, continuous relationships, avoiding the need to repeat their story. Moreover, being referred by a trusted GP could help lower the threshold to seek peer support. Accessibility was defined by practical factors such as nearby locations, extended opening hours, low costs, and the absence of formal requirements like referrals or intake procedures. Immediate access, flexible service options (including online access), and the option to access peer support without a prior appointment were especially important for youth, offering a contrast to the limited time and structure of traditional care.

#### 3.3.2. Peer support workers

All stakeholder groups emphasized the importance of peers having strong interpersonal qualities such as empathy, openness, and a non-judgmental attitude. Peer support workers themselves highlighted the need for self-awareness, mutual respect, and the capacity to inspire hope, acknowledging the responsibilities inherent in their role despite its informal nature. Youth described conversations with peers as more natural, enabling them to speak at their own pace. Although similarity in age and background were valued, the ability to truly listen was seen as more important. Professionals expressed concerns about potential negative influences of peer support workers on youth, such as projecting negative outlooks or undermining professional care. They emphasized the importance of peer workers maintaining a positive and supportive attitude, noting that having experienced hardship themselves can, in some cases, lead to bitterness, which may negatively affect the support they provide.

**Lived experience.** Lived experience was widely considered a defining characteristic of peer support, distinguishing it from traditional healthcare. Youth valued connecting with peers who had faced similar challenges - not for identical experiences, but for shared emotional understanding and vulnerability. This reduces feelings of isolation and stigma. Peer support workers shared this view, seeing lived experience as helpful but not necessary for meaningful connection. Professionals, however, tended to view lived experience as a formal requirement for being able to connect with help-seeking youth. Across groups, peer support was considered most effective when provided by individuals who had reached a level of stability and could serve as hopeful, relatable role models for recovery.

**Training and supervision of peer support workers.** All stakeholders emphasized the need for balanced training that preserves the authenticity of lived experience while ensuring safe and effective support. While some youth favored peers with a level of education comparable to professionals, others preferred minimal training, fearing extensive training would reduce accessibility and shift peer support too close to traditional care. Most participants favored a middle ground: training that promotes safety, encourages reflective practice, and helps peer support workers manage boundaries and avoid overreliance on personal experiences while preserving the authenticity of the peer role. Professionals, and to a lesser extent youth, stressed that training is essential to prevent overreliance on personal experiences, manage risks, and maintain boundaries.

Supervision was considered essential, especially when peer support workers were still navigating their own challenges. Professionals emphasized this need most strongly, though other stakeholder groups also recognized its importance in mitigating the stressors inherent to the peer support environment. Professional supervision, including readiness assessments, post-session debriefings and offering aftercare, helps safeguard both peers and youth while maintaining the informal character of peer support. Additionally, a buddy system - where two peer support workers collaborate to support one help-seeking individual - was suggested to enhance safety, validation, and quality of care.

#### 3.3.3. Form of peer support

Youth consistently stated that their preferred form of peer support depended on both personal factors such as personality, copings skills, and their specific issues and needs, as well as situational factors like their support network, symptom severity, and stressful circumstances in their personal lives. They expressed a clear need for diverse, flexible options to match changing needs.

Walk-in locations were generally favored over scheduled appointments, though the lack of continuity with the same peer worker was seen as a drawback. Most preferred consistent contact with one or a few peers to build trust, unless the goal was simply to ventilate their feelings. Individual sessions were generally preferred over group settings, though the latter was valued for peer learning despite concerns about negative peer influence. Opinions regarding group leadership were neutral, with most youth indicating no strong preference between a professional or a peer. Online forms of peer support, such as online chat platforms, video calls, or phone support, were generally unpopular. Peer support workers also highlighted the importance of live interactions, noting that the ability to observe body language and tone of voice enhances their capacity to understand and respond effectively to youth’s needs. A form of peer support in which a professional pairs young people with similar backgrounds or experiences, was well-received by some, though strict supervision was seen as essential for safety and well-being in such models.

### 3.4. The role of peer support in primary healthcare

All stakeholder groups agreed that peer support adds a valuable, previously unavailable dimension to the mental healthcare system. Youth primarily valued it as a way to address gaps in their social networks and relieve pressure on personal relationships. They appreciated the authentic understanding that comes with shared experiences. Peer support workers agreed, highlighting how lived experience fosters connection and empowerment, and reduces isolation. Professionals saw peer support as a cost-effective means of normalizing and deprofessionalizing mental healthcare. All stakeholder groups recognized peer support as a complementary form of care—valuable for bridging waiting periods and addressing milder mental health issues, but not a replacement for specialized treatment in severe cases.

#### 3.4.1. Cooperation between peer support and primary care

All stakeholder groups highlighted the need for communication and cooperation between peer support and primary care. Professionals emphasized structured referral pathways and comprehensive knowledge of peer support organizations, including details on local initiatives, volunteer qualifications, intake processes, and supervision structures. They expressed greater willingness to refer patients when a healthcare professional was present within the peer support setting and when they had personal familiarity with the staff at the facility. Peer support workers stressed clear communication channels with primary care providers to facilitate appropriate referrals and integrated support. However, they also voiced concerns about maintaining confidentiality and the low-threshold nature of peer support, warning against excessive information sharing that could harm trust. Youth had mixed views as some preferred anonymity, while others accepted limited communication between peer support workers and their GP, provided they retained control over the information shared. Professionals supported information exchange only with explicit consent but remained cautious due to peers’ often voluntary, non-regulated status.

## 4. Discussion

The aim of the current study was to investigate how youth with mental health issues, peer support workers, and primary care professionals perceive youth’s needs, the key elements of peer support, and its potential integration into primary healthcare. Overall, all stakeholder groups acknowledged the potential value of peer support within the primary healthcare system. Peer support offers low-threshold, de-stigmatizing support that may reduce the burden on primary and specialized care. Participants saw it as a complementary option, particularly helpful to bridge waiting times or in the absence of a supportive network. Professionals appreciated its accessibility and potential cost-efficiency but stressed its limitations for more severe conditions.

Effective collaboration between peer support services and primary care remains complex yet crucial. It requires structured referral pathways and clear communication channels, while prioritizing confidentiality and autonomy. These findings align with previous research identifying barriers to integration, such as condescending attitudes, bureaucratic language, and a lack of role clarity and guidelines (De Beer et al., 2023). Effective collaboration can only be meaningfully established when it responds to the core needs of youth, while also reflecting the insights and responsibilities of professionals and peer support workers - laying the groundwork for a shared, youth-centered approach to care.

Central to youth’s needs was the desire to be heard, validated, and approached without judgment. They valued autonomy and preferred services that adapt to their fluctuating needs, personalities, and personal circumstances. They appreciated the freedom to speak at their own pace in the absence of clinical pressure. Both professionals and peer support workers recognized these needs and highlighted the role of trust, continuity, and flexibility in support relationships. Where traditional care may feel formal or rushed, peer support offers a safe, informal space grounded in lived experience. While professionals aim to validate young people’s stories, peer support uniquely offers emotional recognition through shared experience, which often goes beyond what the social network or healthcare system can provide. Peers use their lived experiences both explicitly, by sharing personal stories to build connections and promote hope, and implicitly, by drawing on their recovery journey to adopt supportive, non-prescriptive approaches (Watson, 2019). This dual capacity to inspire hope and offer practical insights through successfully navigated challenges, while balancing informal care with professional safeguards, underscores peer support’s distinctive and valuable role within the mental healthcare system.

However, stakeholder views differed on what qualifies as relevant lived experience. While youth valued shared emotional challenges over identical experiences, peer workers were even more flexible, seeing lived experience as helpful but not essential. In contrast, professionals viewed lived experience as a formal requirement for being able to connect with help-seeking youth. These differences have implications for peer recruitment, training, and expectations. Peer support organizations should therefore clearly communicate the value of diverse forms of lived experience and provide transparent information about what peer support entails. Maintaining flexibility in volunteer recruitment to include a wide range of lived experiences is crucial to ensure accessibility, broaden the volunteer pool, and reduce barriers to expanding peer support services.

Limitations of the current study include participants’ varying familiarity with peer support, which likely introduced differences in how they interpreted and discussed peer support services. This may have affected the depth and internal consistency of the thematic findings, as those with firsthand experience could reflect on direct interactions and experiences rather than abstract or hypothetical understandings. While the informational leaflet provided to participants aimed to offer neutral and objective information about peer support to inform those with limited knowledge, there remains a risk of potential priming effects. Providing this material prior to the interviews may have inadvertently influenced participants’ perceptions or responses before they had an opportunity to articulate their own understanding and experiences. Such pre-interview exposure could have introduced demand characteristics or social desirability biases, particularly among participants without prior experience with peer support services. Additionally, some professionals had limited experience with youth mental health, possibly reducing the depth of their insights. Likewise, some youth confused care providers (e.g., POH-GGZ vs. psychologists), which may have affected response accuracy despite efforts to clarify during interviews. Future studies could provide clearer and more meaningful insights by only focusing on individuals with direct experience with peer support, thereby reducing potential confounds related to unfamiliarity or prior assumptions shaped by pre-interview informational materials.

Another limitation concerns the composition and size of the sample. The study included a disproportionate number of university students, likely reflecting local demographics and recruitment strategies. Together, the limited sample size and overrepresentation of students may constrain the transferability of the findings to more diverse youth populations, including underrepresented groups. Future research should therefore aim to broaden the participant pool across socioeconomic and cultural backgrounds to ensure that the perspectives of underrepresented groups are included, thereby strengthening the external validity of conclusions. Such research should also explore subgroup-specific needs among youth and examine how these may evolve over time, to better inform tailored approaches to peer support.

Future research should also prioritize the integration of peer support within primary healthcare, with particular emphasis on strengthening collaboration and communication. While all stakeholders acknowledge the value of peer support, greater knowledge exchange is needed to align expectations and practices. Such insights are crucial for building a responsive mental healthcare system that effectively combines peer support with primary and specialized care.

## 5. Recommendations for effective collaboration between peer support services and primary care

Based on the combined insights from all stakeholder groups, several key recommendations emerge for enhancing the collaboration between peer support services and primary care. Our results highlighted the need for clear communication channels between primary care and peer support providers, structured training and supervision, role clarity, and tailored options in peer support.

### 5.1. Recommendation 1: provide relevant information and create clear communication channels

Both youth and professionals reported limited knowledge about peer support, creating a dual barrier to the accessibility of these services. Young people struggle to identify appropriate care options and tend to rely on more traditional services. Simultaneously, professionals hesitate to refer due to little familiarity with peer support, resulting in missed opportunities for complementary care. Providing practical, accessible information on local peer support initiatives empowers youth and equips professionals to guide them effectively. Structured communication channels - such as regular updates and transparent documentation of safety and supervision protocols - between peer support providers and primary care practices can strengthen trust and cooperation. Additionally, our data revealed a disconnect in views on information responsibility: young people expect professionals to provide clear information on support options, while some professionals place this responsibility on youth. To address this mismatch, primary care professionals should be equipped with standardized information packages, while peer support services should provide several structured access pathways, enabling youth to independently reach out for support or be referred by primary care providers, ensuring all interactions occur within a structured and supervised setting.

### 5.2. Recommendation 2: provide professional training and supervision that balances safety and authenticity

To preserve the unique characteristics of peer support while ensuring safety, peer support workers require ongoing supervision and comprehensive training. This includes skills such as risk assessment, boundary-setting, conversational techniques, conflict management, recovery principles, and motivational interviewing, while also providing a foundational understanding of mental health disorders (Gopalan et al., 2017; O’Brien et al., 2016; Simmons et al., 2020). However, excessive formalization may compromise the unique, experience-based value of peer support, potentially undermining its meaningful impact (Vandewalle et al., 2016). A balanced approach is crucial to maintain authenticity while meeting safety and care standards.

### 5.3. Recommendation 3: establish role clarity to enhance collaboration between primary care professionals and peer support workers

While the diverse and flexible roles of peer support workers are advantageous, they can also lead to confusion regarding their objectives and value, which in turn undermines both effectiveness and cooperation (Mancini, 2018). Clear role definitions, supported by comprehensive guidelines, joint training, and co-production, are essential for enhancing mutual understanding and integration of peer support services and primary care (Ahmed et al., 2015; De Beer et al., 2024). Establishing role clarity can foster trust, improve collaboration, and ensure young people receive coordinated and tailored mental health support.

### 5.4. Recommendation 4: enhance flexibility and tailored options in peer support

Youth demonstrated diverse and fluctuating preferences for peer support, influenced by factors such as symptom severity, life events and social context. Static healthcare models may struggle to accommodate this variability, underscoring the importance of a flexible, needs-based approach. Expanding the range and adaptability of peer support options can improve accessibility, reduce barriers to help-seeking, and enable more tailored referrals by primary care professionals.

## 6. Conclusion

In conclusion, the current study provides valuable insights into the perspectives of youth, peer support workers, and primary care professionals regarding peer support services. Central to the findings is the recognition of youth’s need for validation, autonomy, and genuine attention, while also highlighting the consensus across stakeholder groups on the importance of accessibility, non-hierarchical relationships, and the need for clear, structured referral systems. Despite these shared understandings, challenges remain in the integration of peer support into primary care. Our findings suggest that successful integration requires careful attention to developing pathways that respect both the informal nature of peer support and the necessity of professional supervision, with an emphasis on information channels, role clarity, and understanding the diverse needs of youth. This approach will facilitate the development of a more inclusive, responsive mental healthcare system that better serves the evolving needs of youth with mental health issues.

## Declaration of generative AI and AI-assisted technologies in the writing process

During the preparation of this work, the author(s) used ChatGPT (OpenAI) in order to improve the English language. After using this tool, the author(s) reviewed and edited the content as needed and take(s) full responsibility for the content of the publication.

## Supplementary Material

Supplementary Material Interview Guides [Supplemental Material A]

Supplementary Material Background Information Provided to Participants [Supplemental Material B]

## Data Availability

The research data supporting this study consist of interview transcripts containing sensitive and confidential information. Due to privacy and ethical restrictions, these data cannot be made publicly available. Further details about excerpts supporting the results of analysis can be obtained from the corresponding author upon reasonable request.
