# Supplementary material for: Bridging peer support and primary care in youth mental health: stakeholder perspectives on needs, key elements and integration challenges
Source: Int J Qual Stud Health Well-being. 2025 Nov 25;20(1):2588933. doi: 10.1080/17482631.2025.2588933 (PMC12667305; doi:10.1080/17482631.2025.2588933)
Supplement: Supplementary Material — Interview Guides [Supplemental Material A] [file ZQHW_A_2588933_SM9060.docx]

**Interview Guides [Supplemental Material A]**

Part of: *Bridging Peer Support and Primary Care in Youth Mental Health: Stakeholder Perspectives on Needs, Key Elements and Integration Challenges*

Authors: Rianne Pellemans-van Rooijen, Mark Spigt, Floor P.M. Koonings, Tom Odink, Verena G. Noort, Thérèse A.M.J. van Amelsvoort & Sophie M.J. Leijdesdorff

This document provides the semi-structured interview guides for youth, peer support workers and professionals used in the study *Bridging Peer Support and Primary Care in Youth Mental Health: Stakeholder Perspectives on Needs, Key Elements and Integration Challenges*. The guides were designed to explore stakeholders’ experiences, perceived needs, and perspectives on integrating peer support within primary care settings for youth mental health.

Abbreviations:

P2P = peer-to-peer support

GP = general practitioner

POH = practice-based mental health professional

**1. Interview Guide Youth**

**Background Information**

Interview No.: Age: Gender:

Living situation: Country of origin: What do you do? (work/study):

**Introduction (knowledge of P2P)**

1. Did you read the background information? Did you gain any new knowledge from it?
2. Where did your previous knowledge come from? Is/was any information missing?
3. How did you first come across peer support?
   Probe: *P2P organization, peer support groups, through a GP/POH, through this research*
4. What does a "peer" mean to you?
   Probe: *a caregiver, someone to talk to, someone with experience, peer of the same age, someone with a similar background, someone with/without professional background*

**Experiences with Healthcare Providers (not the main focus of the research, but...)**

1. What kind of problems prompted you to seek help?
2. What kind of help did you receive?
   Probe: *which healthcare providers? Psychologist, GP, POH, P2P?*
3. Did this experience influence your expectations of mental healthcare?

**Experience with P2P**

1. What made you go to peer support?
   Probe: *issues, recommended by a provider, lack of options, different from regular care, practical assistance*
2. What motivates you to use P2P support specifically?
   Probe: *easy access, reduced stigma, social contact, role model*
3. Why does it or does it not resonate with you?
   Probe: *personal characteristics, issues, needs, personal traits, age group*
4. What were your expectations of it?
5. Has your opinion changed after receiving P2P support?
6. How did you feel after the interaction?
   Probe: *compared to primary care, mental health services, friends/family*

**No Experience with P2P**

1. Have you ever considered it?
2. Why would it or would it not be suitable for you?
   Probe: *personal characteristics, issues, needs, personal traits, age group*

**Expectations of P2P**

1. What is important to you in peer support?
   Probe: *waiting lists, intake, accessibility, informal sources of information*
2. How do you envision the role of a peer?
   Probe: *a caregiver, someone to talk to, someone with lived experience, peer of the same age, someone with a similar background, someone with/without professional background, supervision*
3. What do you consider important in peer interactions?
   Probe: *professionalism, dynamics, boundaries*
4. How do you imagine someone with lived experience?
   Probe: *how close should the experience be?*
5. What does P2P need to match your desires/needs?

**Characteristics of P2P**

1. What do you see as the pros and cons of different aspects within P2P support?
   Probe: *individual vs. group, professional peer vs. layperson, embedded professional? Lived experience? Single conversation or longer process? Scheduled or walk-in? Online vs. face-to-face?*
2. Which characteristics do you find important and which not? And why?
3. Do you think your considerations align with those of others?

**Choices for Care, GP/POH**

1. Why did you choose to seek care from or not from the GP/POH?
   Probe: *fit with your needs or concerns, waiting lists, connection with the provider*
2. And the choice for P2P?
3. What do you expect the GP/POH to do differently from P2P?
4. What is important in care for you?

**Communication between Primary Care and P2P**

1. Does the GP/POH know you're using P2P? Do you discuss it with them?
2. Is there a need for (more) communication between the GP/POH and P2P provider?
   Probe: *what would it entail? Content-related or just knowing you're using P2P, at intake or throughout the process, referral letter, proactive communication between GP/POH and P2P*

**Collaboration between Primary Care and P2P**

1. Would you find it desirable for the GP/POH and P2P to collaborate?
   Probe: *similar to psychologist or POH*
2. Would the level of integration make a difference?
   Probe: *co-facilitation, joint treatment, separate entity*

**Miracle Question (Opinion on P2P)**

1. How would P2P support look if it were up to you?
2. If the current mental health system worked perfectly, what role would P2P support play?

**Check**

What is someone seeking: *(issue - conversation, practical help, social contact, etc.)*

Issues: *(anxiety, mood, trauma, etc.)*

How often have they been in contact with P2P/the GP about this (number of conversations):

For what issues did you seek help? What kind of help did you receive and for how long?

**Concluding**

- Have all discussion points been covered? From both the interviewer and the participant?
- Verify information
- Explain the gift card that will be sent

**2. Interview Guide Peer Support Workers**

**Background Information**
Interview no: Position: volunteer @ease Location:
Gender: Age: Education level:
**Introduction**
 1. What is peer support?

2. Which experiences do you have with peer support? How did you initially come into
 contact with peer support?
 Probe: *experiences before receiving and offering peer support, if yes, which effect did
 this have on you?*

3. Which other providers of peer support do you know of?
**Expectations of P2P**

4. How do you envision the role of a peer? What do you consider important in peer
 interactions?
 Probe: *a caregiver, someone to talk to, someone with lived experience, peer of the same
 age, someone with a similar background, someone with/without professional
 background, supervision*

5. What do you expect adolescents need from P2P support?

6. What kind of attitude is expected from a peer? What kind of interaction/dynamic is
 expected?
 Probe: *(in)formal*, *boundaries, role, professionalism*

7. What is the added value of someone with lived experience?
 Probe: *how close should the experience be?*

8. What do you consider important in peer interactions?
 Probe: *professionalism, role clarity, range of expertise, boundaries, confidentiality,
 privacy*

9. What kind of supervision/monitoring is offered to peer supporters? And is supervision
 desired?
 Probe: *role of supervisor/peer, who is responsible?*

10. Are there any thresholds for adolescents to find help? If yes, what are these
 thresholds? **Characteristics P2P**

11. What do you see as the pros and cons of different aspects within P2P support?
Probe: *individual vs. group, single conversation or longer process, embedded | professional or not, professional peer vs. layperson, lived experience, scheduled or walk-in, online vs. face-to-face?*

12. Which aspects are important for you as a volunteer/peer supporter?

13. Which aspects are important to adolescents?

**Subgroups Adolescents**

14. Are there different types of subgroups of adolescents that make use of P2P? If yes, is
 P2P suitable for all these groups?

15. Why is P2P better suitable for some types of adolescent groups?

**Miracle Question**

16. How would P2P support look if it were up to you? And what would it look like if it was
 up to the adolescents?

17. If the current mental health system worked perfectly, what role would P2P
 support play?

**Collaboration**

18. Would it be desirable for the GP/POH-GGZ and P2P to collaborate? If yes, how
 would this collaboration operate?

**Communication**

19. Are you informed of any adolescents who receive care from a GP/POH-GGZ? If yes, is
 there a need for (more) communication between the GP/POH and P2P provider?
 Probe: *what would it entail? What attitude do you think the adolescents have on this?
 Which influence does this have on the low threshold of P2P?*

**Concluding**

- Have all discussion points been covered? From both the interviewer and the participant?
- Verify information

**3. Interview Guide Professionals**

**Background Information**

Interview no.: Role (GP/POH-GGZ): Gender:

Practice: Experience with P2P support: yes/no

**Introduction (knowledge of P2P)**

1. Have you read the information? Does the framework we provided align with your own knowledge or experiences?
2. Where does your prior knowledge come from? Have you encountered this concept in the medical field?
   Probe: *experience with P2P organizations, continuing education, formal training, professional journals, literature*
3. Are you familiar with providers?
4. Is there a need for more information about P2P support and providers, and if so, from whom should this information come?
   Probe: g*uidelines, umbrella organizations, continuing education, formal training, professional journals, literature*
5. What is your understanding of P2P support?

**Experiences with P2P Support as a Healthcare Provider**

1. Have any of your patients ever used P2P support? Whose initiative was it?
   Probe: *your recommendation, patient-initiated, or other provider? Type of support? One-time or ongoing?*
2. What impact did it have on the patient’s care trajectory? Did it contribute positively? What advantages or disadvantages did you observe?

**Expectations of P2P Support (Conceptual, Based on Expert Opinion)**

1. How do you define a peer? What is important?
   Probe: *healthcare provider, someone to talk to, lived experience, same age group, similar background, with or without professional experience*
2. How should a peer conduct themselves? What dynamics are expected?
   Probe: *(in)formality, boundaries, role, professionalism*
3. How do you envision someone with lived experience? What added value do they bring?
   Probe: *experienced expert vs. someone with personal experience; how close should the experience be?*
4. What do you consider important in P2P support?
   Probe: *professionalism, role clarity, diverse expertise, boundaries, confidentiality, privacy*
5. What supervision or monitoring is desirable?
   Probe: *role of supervisor/peer, where responsibility lies*
6. Do you have any concerns about aspects of P2P support?
7. What do you expect your patients/young people need from P2P support?

**Characteristics of P2P Support (Conceptual)**

1. What do you see as the advantages and disadvantages of different features of P2P support?
   Probe: i*ndividual vs. group, short conversation vs. longer program, professional involvement, professional peer vs. layperson, lived experience, scheduled vs. voluntary, online vs. face-to-face*
2. Which aspects are important to you?
3. Which aspects are important to your patients?
4. Does what is offered lead to different considerations when recommending it to patients?

**Patient Subgroups**

1. Does peer support suit every patient group? Are there specific subgroups for whom P2P is particularly suitable or unsuitable?
2. Do different P2P features meet different needs? Which ones and why?

**Communication**

1. Are you aware of any patients who have used P2P support? Do they discuss it with you?
2. Do you currently communicate with providers about your patients? To what extent?
3. Is there a need for (more) communication with providers or peers?
   Probe: *what might this look like? Similar to communication in mental health or other healthcare organizations? Referral letters, intake, or the entire care trajectory?*

**Collaboration**

1. Would you consider it desirable to have (more) collaboration between primary care and P2P providers?
   Probe: s*imilar to collaboration in mental health or other healthcare organizations?*
2. Would the degree of integration make a difference to the patient’s care trajectory?

Probe: *co-facilitation, joint treatment, or separate/independent organization?*

**Miracle Question (Opinion on P2P)**

1. What would P2P support look like if it were up to you? And if it were up to your patients?
2. If the current mental health care system were functioning perfectly, what role would P2P support play?

**Concluding**

- Have all discussion points been covered? From both the interviewer and the participant?
- Verify information
