# Supplementary material for: Bridging peer support and primary care in youth mental health: stakeholder perspectives on needs, key elements and integration challenges
Source: Int J Qual Stud Health Well-being. 2025 Nov 25;20(1):2588933. doi: 10.1080/17482631.2025.2588933 (PMC12667305; doi:10.1080/17482631.2025.2588933)
Supplement: Supplementary Material — Background Information Provided to Participants [Supplemental Material B] [file ZQHW_A_2588933_SM9061.docx]

**Background Information Provided to Participants [Supplemental Material B]**

Part of: *Bridging Peer Support and Primary Care in Youth Mental Health: Stakeholder Perspectives on Needs, Key Elements and Integration Challenges*

Authors: Rianne Pellemans-van Rooijen, Mark Spigt, Floor P.M. Koonings, Tom Odink, Verena G. Noort, Thérèse A.M.J. van Amelsvoort & Sophie M.J. Leijdesdorff

This document provides a brief background description of the concept *peer support*. It was shared with participants prior to the interview to ensure a basic understanding of the term and to support reflection on their experiences and perspectives. The purpose of this material was not to define the concept exhaustively, but to provide a shared reference point for the discussion.

**Background information on peer support study**

During the interview we’ll talk about, among other things, preferences and needs of young people when seeking and receiving mental healthcare, and how this aligns with the various characteristics of peer support. On this page, you will find some background information about this. We ask you to review it briefly so that even when participants have little or no knowledge and experience with peer support, this topic can still be discussed. This information is intended only as an aid; participants are not expected to study it extensively, and there will be no quiz.

*Peer* is an English term that doesn't translate well to Dutch. It means someone from a similar background. This can be in terms of age, education, or life experience.

*Peer support* involves people who have gone through similar things helping each other. Based on their experience with certain situations, illnesses, or problems, a peer can assist someone currently going through something similar. They don't provide professional advice but can offer tips, share what helped them in that situation, offer comfort, or simply engage in conversation.

We are researching peer support in mental healthcare, but peer support can be used in other contexts as well. For example, in grief, serious or chronic illnesses like cancer or diabetes, or traumatic events.

There are many different ways to offer or use peer support. Below, we have listed some characteristics of peer support that can vary:

- Having a **face-to-face** conversation, which can take place at home, in the doctor’s or psychologist’s office or at the hospital, or **online** peer support through platforms like online forums, chat services, or WhatsApp.
- Peer support can be offered **individually** or in **group therapy**. In individual peer support one person seeks help and interacts with a peer. In group therapy several people seek help, either with or without the guidance of a peer. If done without a group leader, the group members are the peers.
- A peer can be a volunteer who has or hasn't followed **training** and helps others in their free time. It can also be someone's job: a **professional** employed by a company or practice who receives a salary and has usually received more education.
- Sometimes, a **traditional professional** like a practice assistant (POH-GGZ), a doctor, or a psychologist is present during the conversation with the peer.
- Peer support can be part of a **treatment plan** from a POH-GGZ, doctor, or psychologist, or it can stand alone.
- Peers often have **experience** with similar problems to those seeking help, but this isn't necessary. Sometimes, it's enough to talk to someone of the same age or someone who can lend a **listening ear.**
